# Conceptual Knowledge Discovery in Databases for Drug Combinations Predictions in Malignant Melanoma

**Published:** 2015

**Authors:** Kelly Regan, Satyajeet Raje, Cartik Saravanamuthu, Philip R.O. Payne

**Affiliations:** a Department of Biomedical Informatics, The Ohio State University, Columbus, OH, USA

**Keywords:** Malignant Melanoma, Knowledgebases, Combination Drug Therapy

## Abstract

The worldwide incidence of melanoma is rising faster than any other cancer, and prognosis for patients with metastatic disease is poor. Current targeted therapies are limited in their durability and/or effect size in certain patient populations due to acquired mechanisms of resistance. Thus, the development of synergistic combinatorial treatment regimens holds great promise to improve patient outcomes. We have previously shown that a model for in-silico knowledge discovery, Translational Ontology-anchored Knowledge Discovery Engine (TOKEn), is able to generate valid relationships between bimolecular and clinical phenotypes. In this study, we have aggregated observational and canonical knowledge consisting of melanoma-related biomolecular entities and targeted therapeutics in a computationally tractable model. We demonstrate here that the explicit linkage of therapeutic modalities with biomolecular underpinnings of melanoma utilizing the TOKEn pipeline yield a set of informed relationships that have the potential to generate combination therapy strategies.

## Introduction

Melanoma is the most deadly form of skin cancer, accounting for nearly 10,000 deaths in the United States in 2014. The incidence of melanoma is rising faster than any other cancer in the U.S., and there were over 76,000 new cases diagnosed in 2014 [1]. The death rate for melanoma patients in the U.S. has remained stagnant for the past 20 years, and less than 20% of patients have shown responses to traditional chemotherapeutic therapies [2, 3]. Cancer-driving *BRAF* mutations (V600E/K) are found in 40-60% of melanoma patient tumors, and BRAF-inhibitor agents, dabrafenib and vemurafenib, have extended median patient survival by 5-6 months [4]. Despite recent advances in targeted therapies, drug resistance remains a significant challenge for melanoma patients. Thus, further work to discover drugs that act synergistically with existing therapies and decrease drug resistance is desirable.

Traditional bench-based approaches for discovering synergistic drug combinations, including high-throughput drug screening, are costly and inefficient [5, 6]. It is estimated that an average of 1 billion dollars and 15-20 years is needed to bring a new drug from the bench to the bedside [7]. Further, 52% of drugs fail during development in phase 1 clinical trials, and only 25% of compounds that enter phase 2 proceed into full phase 3 clinical studies [8]. Biomedical informatics methods may offer more efficient and efficacious approaches for identifying synergistic drug combinations. Several computational approaches to optimize the drug discovery process have been proposed that involve modeling of structural, biochemical and biophysical properties [9].

In this study, we aim to computationally identify possible drug combinations to act synergistically with BRAF inhibitor therapies using a knowledge-anchored approach. The use of Conceptual Knowledge Discovery in Databases (CKDD) methods provides a potential means to accelerate hypothesis generation and recapitulation of known relationships between combinations of database entities. We have previously shown that a model for *in-silico* knowledge discovery, Translational Ontology-anchored Knowledge Discovery Engine (TOKEn), is able to generate valid relationships between bimolecular and clinical phenotypes in the context of large-scale, chronic lymphocytic leukemia datasets [10].

**Figure 1:**
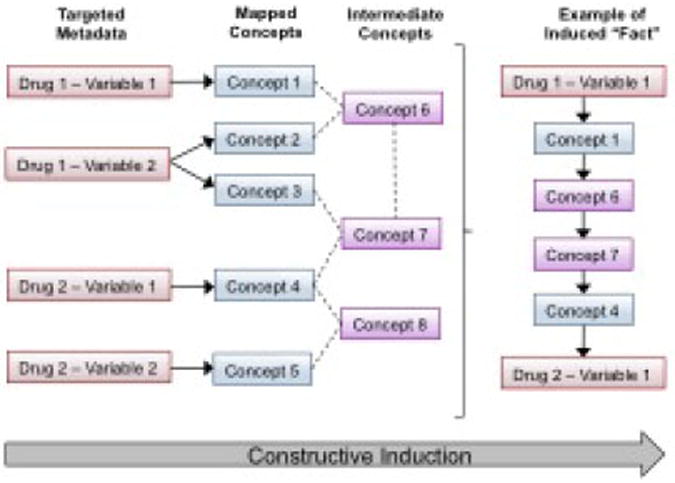
Constructive induction of conceptual facts between distinct drugs. Mapping between database elements of targeted metadata to corresponding ontology concepts are utilized to induce “facts” among database elements, in this case, distinct drugs. Concepts 6 and 7 represent intermediate concepts not mapped to an original drug database element that define a higher-order transitive path that begins and terminates with drug database elements.

Knowledge discovery in databases represents a type of conceptual knowledge engineering method used to characterize relationships among distinct elements contained within a database [11]. Domain-specific knowledge collections, such as ontologies, are commonly used during knowledge discovery to augment meta-data contained in the targeted database schema. This overall approach is the basis for constructive induction, a type of knowledge discovery in databases (Figure 1). The constructive induction process generates conceptual knowledge constructs, otherwise referred to as induced “facts,” that are defined by data elements and the semantic relationships that link them. Resulting conceptual knowledge constructs may be used to generate potential hypotheses about relationships between distinct data elements. Previous evaluation of the TOKEn method demonstrated its validity and “meaningfulness” according to domain experts [10]. Here we present the first application of TOKEn aimed at identifying drug combinations in malignant melanoma.

## Methods

**Figure 2:**
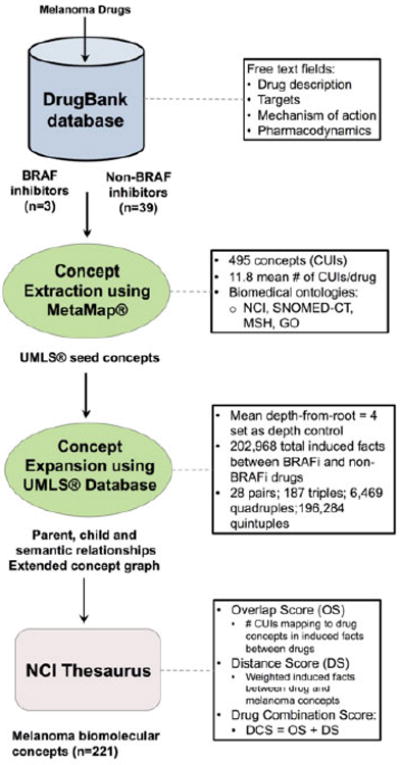
Overview of TOKEn and DCS workflow

The TOKEn workflow has been previously described [10]. The overall workflow specifically applied in this study is shown in Figure 2. We obtained 42 FDA-approved and investigational “melanoma” drugs from DrugBank (version 4.1) [12]. DrugBank is a comprehensive database that includes chemical, pharmacological and pharmaceutical drug information as well as sequence, structure and pathway information regarding drug targets into more than 200 data fields per therapeutic agent. Relevant data fields pertaining to biomolecular foundations of drug action were selected, including “description,” “mechanism of action,” “pharmacodynamics” and “targets.”

We developed an automated method to map selected DrugBank database fields containing free text to concepts within the Unified Medical Language System (UMLS), and selected those concepts belonging to the NCI, SNOMED-CT, MSH and GO ontologies due to their broad coverage, including concepts related to drug features and actions. Similarly, a set of semantic types was heuristically defined to generate hypotheses targeted to drugs. Mapped entities were subsequently reviewed manually for accuracy and relevancy for mechanistic underpinnings of therapeutic agents. We obtained UMLS Metathesaurus associations from the previously curated set including parent, child and semantic relationships that were refined by subject matter experts to filter those relationships to be most meaningful for relating biomolecular and phenotypic concepts [10]. We determined that these heuristics generated in the original TOKEn study to be sufficiently generalizable for our purposes. We set search space optimization controls for the constructive induction method by calculating the shortest path depth-from-root of the ontology concepts selected and used them to annotate concepts as an indicator of concept granularity.

The UMLS MRHIER source file indexes all unique hierarchical paths determined by the source vocabulary as strings of distinct atoms from a particular concept to the UMLS root concept. Using this file, the minimum distance to the root was calculated for each UMLS concept corresponding to the source vocabularies. For each concept unique identifier (CUI), we set the ‘minimum distance to the root’ equal to the minimum number of elements in the corresponding path-to-root fields. The average depth of the ontology concepts that were mapped from the initial DrugBank data elements was found to be 4 “steps” from the UMLS root. We generated induced “facts” (Figure 1) using the graph-theoretic constructive induction algorithm previously described [10]. Traversal paths for drug combinations initiated at concepts associated with BRAF inhibitor drugs (vemurafenib, dabrafenib, PLX-4032) and terminated at those associated with the remaining 39 non-BRAF inhibitor drugs in the set. The algorithm avoids cycles by preventing the inclusion of duplicate concepts within a single traversal path. We constrained all concepts included in the induced “facts” to be at a depth equal to or greater than the minimum of the initial and terminal concepts. Pairs include direct relationships between drug-related concepts, while triples and quadruples include 1 and 2 intermediate concepts, respectively.

In order to prioritize drug relationships generated via the TOKEn method, we incorporated a novel ranking method according to their relatedness to melanoma pathogenesis. We obtained 663 “melanoma” concepts within the NCI Thesaurus (version 14.07d), and of those we used 221 concepts related to biomolecular properties of the disease. For example, we excluded tissue-level diagnoses or other high-level disease terms (uveal melanoma, acral melanoma, etc.). We further applied the TOKEn method to generate relationships that initiated with these NCI melanoma-associated concepts and terminated with concepts derived from the 42 DrugBank melanoma-associated therapies.

In order to rank BRAF inhibitor and non-BRAF inhibitor drug pairs generated via TOKEn, we calculated a Drug Combination Score (DCS) using the sum of two metrics for non-BRAF inhibitor drugs. Since BRAF inhibitors are expected to have the same degree of relatedness to melanoma pathogenesis, dabrafenib, vemurafenib and PLX-4032 were weighted equally. For each non-BRAF inhibitor drug, we summed: the Overlap score, the number of concept unique identifiers that mapped directly to drug concepts and those intermediate concepts derived from the induced facts between drugs that overlapped with the melanoma-associated set of CUIs; and the Distance score, the number of induced fact relationships between drug and melanoma concepts that were also weighted to the number of hops between initial and terminal concepts, where proximal relationships (i.e. fewer hops) were given a higher weight. Resulting Drug Combination Scores (DCS) were rank-ordered from highest to lowest for each non-BRAF inhibitor drug and final paths to concepts BRAF inhibitor. Log-2 transformed values for scores are reported.

## Results

### Concept identification and constructive induction

The 42 melanoma drugs indexed in DrugBank were mapped to UMLS concepts. Following manual review, a total of 495 drug-related UMLS concepts were identified for this study. The mean and median number of unique concepts per drug were 11.8 and 10.5, respectively. The BRAF inhibitor drugs (n=3) and non-BRAF inhibitor drugs (n=39) mapped to 23 and 300 unique concepts, respectively. The total numbers of induced facts in this study are listed in Table 1. For the total number of pairs, triples, quadruples and quintuplets, 28, 187, 6,469, and 196,284 concepts were anchored to a BRAF inhibitor. A total of 202,968 induced facts between BRAF inhibitor and non-BRAF inhibitor drugs were generated. In Table 2, examples of induced traversal paths between BRAF inhibitor drugs (vemurafenib, dabrafenib, PLX-4032) and non-BRAF inhibitor drugs are shown.

### Drug combination scoring of induced facts

The FDA-approved anti-melanoma drug combination of trametinib and dabrafenib is evidenced here (Table 2) by the recognized relationship between the MAP2K1 and BRAF proteins in the MAPK signaling pathway and phosphorylation of its constituent proteins in melanoma tumors [13]. Furthermore, the combination regimen of trametinib and dabrafenib was recently approved by the FDA for use in melanoma patients with BRAF V600E or V600K mutations. Although PI-88 is an investigational drug not currently approved by the FDA, we show here evidence that it may support inhibition of tumor angiogenesis in combination with other BRAF inhibitors.

We implemented the DCS scoring metric to rank proposed BRAF inhibitor and non-BRAF inhibitor drug combination pairs predicted by the TOKEn algorithm (Table 3). The number of unique mapped concepts for the BRAF inhibitors dabrafenib, PLX-4032 and vemurafenib were 16, 3, and 14, respectively. Importantly, all three BRAF inhibitors shared the common set of concepts “BRAF gene,” “Proto-Oncogene Proteins B-raf,” and “Phosphotransferases” that were identified as initial concepts in all induced relationships that terminated with those associated with non-BRAF inhibitor drugs. Due to this congruency among concepts and the common therapeutic action of inhibiting BRAF protein activity, BRAF inhibitors were weighted equally in our scoring algorithm.

The Distance score component of the DCS was calculated over 69,856 induced facts between non-BRAF inhibitor and melanoma concepts. The values of non-zero log-2 transformed DCS ranged from 4.25 (AS1409) to 28.83 (AGRO100). In principle, the Overlap score emphasizes the direct relationships between drug and melanoma concepts (e.g. drug targets representing melanoma genes), with the tradeoff of possibly severely limiting potential drug-disease connections. Conversely, the Distance score emphasizes indirect relationships, or induced facts, between drug and melanoma concepts. Of note, the Overlap scores and Distance scores were significantly correlated among all non-BRAF inhibitor drugs (Pearson = 0.46, p<0.0031). Thus, these component metrics can be viewed as complementary when used to rank drug combination predictions. Others have shown that distance-based metrics can be used to determine the similarity between drugs based on conceptual knowledge for drug repurposing applications [14-17]. Future work will assess the individual contributions of these metrics in validated drub combination relationships.

## Discussion

In this study, we present the first application of the TOKEn method for *in-silico* knowledge discovery to database-derived melanoma drug-disease relationships. We developed a novel ranking metric for TOKEn-generated hypotheses for BRAF inhibitor (n=3) and non-BRAF inhibitor (n=39) drug combinations in melanoma. We found 202,968 unique relationships linking BRAF inhibitor drugs to 30 out of 39 non-BRAF inhibitor melanoma drugs that were indexed in DrugBank. AGRO100 was the highest ranked non-BRAF inhibitor drug for use in combination with BRAF inhibitors. AGRO100 is an experimental anticancer agent that acts as an aptamer and inhibits nucleolin, a protein that is uniquely expressed on the surface of tumors cells.Results from a phase I study of AGRO100 in patients with advanced cancers showed that half of the patients enrolled had stable disease with no toxic effects observed in any patient [18]. Further clinical development of AGRO100 resulted in the modified AS1411 agent, which has shown subsequent success in clinical applications [19, 20]. Interestingly, AS1411 was shown to reduce levels of a specific subset of miRNAs through its actions on nucleolin, including miR-21, miR-221, miR-222 and miR-103. These miRNAs are causally involved in breast cancer initiation, progression and drug resistance, and are also well known melanoma-associated miRNAs. Of note, all of the interferon-based therapies, with the exception of natural alpha interferon, were highly ranked according to their DCSs (rank 2-4).

Recently, combinations of BRAF inhibitors and immunotherapies have shown improved efficacy treating melanoma tumors and are being investigated in clinical trials [21]. Trametinib, a MEK inhibitor, is part of an FDA-approved combination with dabrafenib, and was ranked 7^th^ according to DCS and 1^st^ according to the overlap score alone. AZD-8330 is an experimental MEK inhibitor, and was ranked 5^th^ according to DCS and 2^nd^ according to the overlap score alone. This application of the TOKEn method has made improvements over the initial iteration by incorporating object-oriented programming and a system by which to prioritize induced facts.

Our study is currently limited by constraining the TOKEn method to canonical knowledge regarding melanoma concepts. The limited information contained within structured databases such as DrugBank may have prohibitively reduced our search space, and may have accounted for failing to recover relationships for 9 out of 39 non-BRAF inhibitor drugs. Furthermore, by limiting this study to known melanoma drugs indexed within DrugBank, we excluded possibilities to evaluate other existing drugs and small molecules that are not currently indicated or investigated in melanoma. Future work will include increasing our search space to other databases targeted to drug and melanoma information, as well as extracting data directly from the literature. We will also conduct future work to validate our findings and approach, including evaluating semantic similarity measures among mapped concepts, using graph-based network modeling methods and formal subject matter expert review.

## Conclusion

We have demonstrated that a model for in-silico knowledge discovery, Translational Ontology-anchored Knowledge Discovery Engine (TOKEn), is able to generate valid relationships between drug and biomolecular phenotypes in the context of malignant melanoma.

## Figures and Tables

**Table 1:** Summary of transitive paths generated at a search depth control of 5 and a distance from root of 4

Number of concepts in induced “facts”	2	3	4	5
Number of unique relationships	5,940	103,540	4,789,356	100,289,621

**Table 2:** Examples of predicted “facts” connecting distinct drugs via triplets

Relationship pattern	Conceptual knowledge constructs
Trametinib → Dabrafenib	MAP2K1 protein – [gene plays roles in biological process] – Serine/Threonine Phosphorylation – [process involves gene] – BRAF gene
PI-88 → Vemurafenib	FGF1 gene – [gene plays role in process] – Angiogenic process

**Table 3:** Top ten ranked non-BRAF inhibtor drugs hypothesized for use in combination with BRAF inhibitor drugs. DCS = Drug Combination Score. Reported DCS, Overlap and Distance scores are log-2 transformed values

Non-BRAF inhibitor drug	DCS	Overlap score	Distance score
AGRO100	28.83	14.46	14.37
Peginterferon-alfa-2a	28.66	13.93	14.74
Interferon alfacon-1	28.45	13.87	14.58
Inteferon Alfa-2b	25.82	11.24	14.58
AZD-8330	25.77	14.68	11.10
Trabectedin	25.33	11.92	13.41
Trametinib	25.02	14.99	10.04
ZEN-012	23.09	11.40	11.70
PI-88	23.01	11.09	11.93
ABT-510	22.31	10.99	11.33
